# Feasibility to Train Older Adults in India to Use Smart-Phone to Increase Participation in Daily Activities

**DOI:** 10.1093/geroni/igaa057.628

**Published:** 2020-12-16

**Authors:** Asish Das, Amit Sethi

**Affiliations:** 1 Manipal Academy of Higher Education, Manipal, Karnataka, India; 2 Occupational Therapy, Pittsburgh, Pennsylvania, United States

## Abstract

Though 60% of 104 million older adults own a smart phone in India, up to 85% of these individuals find challenging to use smart-phone during daily activities. This is problematic because inability to use smart-phone limits their ability to participate in daily activities such as, managing their health, paying bills, shopping and communicating with their loved ones, resulting in social isolation and dependence on others. The purpose of this study was to examine the feasibility of training older adults to use smart-phone to increase participation in daily activities. This preliminary study was conducted over two phases. In phase-I, we used the principles of stakeholder engagement to interview 12 older adults to identify the barriers associated with the use of technology using a brief survey. Based on the results of Phase I, we implemented a single education and training session for 42 participant (> 60 years) in using smart-phone based applications in Phase II. Older adults were trained to use smart-phone based applications to manage money using banking apps, pay bills, shop, manage health to monitor vitals and medications and communicate with their loved ones. All participants rated their satisfaction with the program, 70% participants showed the ability to use the applications independently. Seventy five percent rated the program extremely useful, and 70% rated it extremely relevant to them. It is feasible to train older adults in using smart-phone based applications. Future studies need to focus on providing follow-up sessions to increase retention and carry-over to increase participation in daily activities.

